# Accurate estimation of short read mapping quality for next-generation genome sequencing

**DOI:** 10.1093/bioinformatics/bts408

**Published:** 2012-09-03

**Authors:** Matthew Ruffalo, Mehmet Koyutürk, Soumya Ray, Thomas LaFramboise

**Affiliations:** ^1^Department of Electrical Engineering & Computer Science; ^2^Department of Genetics; ^3^Center for Proteomics and Bioinformatics, Case Western Reserve University, Cleveland, OH 44106, USA

## Abstract

**Motivation:** Several software tools specialize in the alignment of short next-generation sequencing reads to a reference sequence. Some of these tools report a mapping quality score for each alignment—in principle, this quality score tells researchers the likelihood that the alignment is correct. However, the reported mapping quality often correlates weakly with actual accuracy and the qualities of many mappings are underestimated, encouraging the researchers to discard correct mappings. Further, these low-quality mappings tend to correlate with variations in the genome (both single nucleotide and structural), and such mappings are important in accurately identifying genomic variants.

**Approach:** We develop a machine learning tool, LoQuM (LOgistic regression tool for calibrating the Quality of short read mappings, to assign reliable mapping quality scores to mappings of Illumina reads returned by any alignment tool. LoQuM uses statistics on the read (base quality scores reported by the sequencer) and the alignment (number of matches, mismatches and deletions, mapping quality score returned by the alignment tool, if available, and number of mappings) as features for classification and uses simulated reads to learn a logistic regression model that relates these features to actual mapping quality.

**Results:** We test the predictions of LoQuM on an independent dataset generated by the ART short read simulation software and observe that LoQuM can ‘resurrect’ many mappings that are assigned zero quality scores by the alignment tools and are therefore likely to be discarded by researchers. We also observe that the recalibration of mapping quality scores greatly enhances the precision of called single nucleotide polymorphisms.

**Availability:** LoQuM is available as open source at http://compbio.case.edu/loqum/.

**Contact:** matthew.ruffalo@case.edu.

## 1 INTRODUCTION

Next-generation genome sequencing (NGS) has quickly become very popular in life sciences because of its utility in efficiently generating high-quality sequence data (Meyerson *et al.*, 2010). Applications of NGS include analysis of gene expression and alternative splicing (RNA-seq) (Wang *et al.*, 2009), DNA–protein interactions (chromatin immunoprecipitation sequencing) (Schmidt *et al.*, 2009), *d*e novo genome sequencing (Li *et al.*, 2009b), metagenomics and identification of genetic variants within and across populations (Alkan *et al.*, 2009). Many computational methods are already available for analyzing genetic variants using NGS data. These variants include single-nucleotide polymorphisms (SNPs) and structural variants such as copy numbers, insertions, deletions, tandem duplications, inversions and translocations. Characterization of such variants is useful in many applications, including genome-wide association studies (Mardis, 2008), identification of driver mutations in cancer (Meyerson *et al.*, 2010) and comparative genomics (Hudson, 2008).

### 1.1 Short read alignment is an important problem

The first step in the detection and analysis of genetic variants is usually the alignment of short NGS reads from an individual's (donor) genome to a reference genome. This task poses significant computational challenges due to the large number of reads (sometimes tens of millions) and the size of many reference genomes (generally on the order of billions). Furthermore, because of sequencing errors, repeats in the reference genome and differences between the donor and reference genomes, accurate mapping of the reads to the genome is not straightforward. In recent years, many software tools have been developed to address these challenges and efficiently and accurately align short reads to the reference genome (Ruffalo *et al.*, 2011). These tools include BWA (Li and Durbin, 2009), SOAP (Li *et al.*, 2009c), Novoalign (Novocraft, 2010) and mr(s)FAST (Alkan *et al.*, 2009; Hach *et al.*, 2010).

### 1.2 Current alignment tools do not return reliable mapping quality scores

**Fig. 1.**
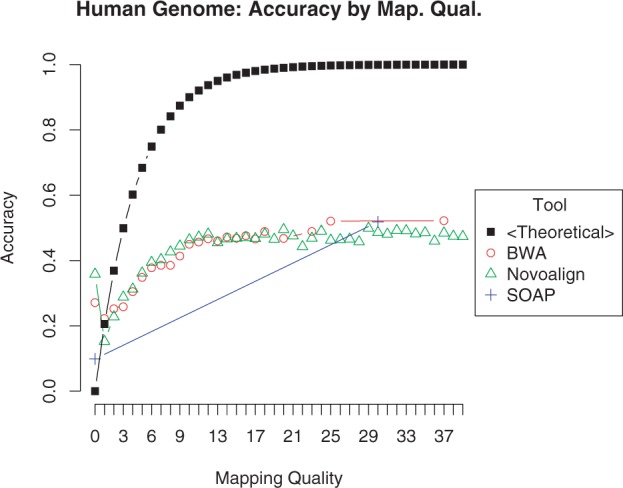
Direct comparison of the theoretical accuracy at each quality score *Q*_m_ against each tool's actual accuracy. The mapping quality *Q*_m_ is defined as the log-scaled probability *P* that the mapping is incorrect: *Q*_m_ = −10log_10_ *P*, giving a theoretical accuracy *A* for each quality score: *A* = 1 − *P* = 1−10^−Q_m_/10^

As the mapping of a read to a location in the reference genome may not be accurate, many alignment tools report a mapping quality score as an indicator of the likelihood that the mapping is accurate. This score is generally estimated by considering various factors, such as the number of base mismatches and the sizes of inserted or deleted regions in the alignment. In principle, the mapping quality score *Q*_m_ reflects the log-scaled probability that the mapping is inaccurate and ranges from 0 (Pr{mapping is inaccurate} = 1, i.e. the mapping is most likely inaccurate) to 40 (40= −10log_10_ Pr{mapping is inaccurate} → Pr{mapping is inaccurate} < 10^−4^, i.e. the mapping is most likely accurate). However, for almost all the state-of-the-art alignment tools, the mapping quality scores do not correlate well with the actual likelihood that a mapping is accurate and is therefore not very useful (Ruffalo *et al.*, 2011). This is illustrated in Figure 1. In the figure, for several alignment tools, the relationship between the mapping quality reported by the alignment tool and the actual mapping accuracy (the fraction of accurate mappings among those that are assigned the respective quality score) is shown. As seen in the figure, alignment tools generally report many accurate mappings with quality 0, and many inaccurate mappings with high-quality scores. We also see that there is very little dynamic range in the mapping qualities; even when tools provide many distinct quality scores, there is little difference in actual mapping accuracy between, e.g. mappings with quality score 15 and mappings with quality score 30. Underestimated mapping quality scores might cause the user to discard many accurate mappings because of the conservative estimate provided by the alignment tool. Overestimated mapping quality scores, conversely, may contribute to false-positive results in variant calling and other types of downstream analysis. These observations call for more accurate assessment of mapping quality for the mappings provided by these tools. Furthermore, some methods, e.g. mr- and mrsFAST, do not report any mapping quality scores, they rather report all possible mappings for a read. For such tools, assessment of mapping quality would be useful as well, as a means for choosing from the multiple possible mappings.

### 1.3 Contributions of this study

In this article, we use a machine learning approach to assess the quality of the short read mappings more accurately than available alignment tools. For this purpose, we first identify the features that are potentially useful in assessing the likelihood that a mapping is accurate. These features consist of read statistics provided by an Illumina sequencer (e.g. base quality) and alignment statistics provided by the aligner (e.g. number of matches, mismatches, deletions, insertions, number of possible mappings and mapping quality score). Subsequently, we simulate NGS runs to generate reads that accurately reflect the characteristics of available sequencers. We use these simulated reads and the mappings provided by the aligner for these reads as training data to fit a logistic regression model that represents the relationship between read and alignment statistics and mapping accuracy. We implement this computational pipeline into a software package, LoQuM (LOgistic regression tool for calibrating the QUality of short read Mappings), which is available as open source at http://compbio.case.edu/loqum/. LoQuM can work with a wide range of alignment tools to run the aligner for the user, compile the mappings returned by the aligner, calibrate the quality of these mappings and return the list of mappings with more reliable mapping quality scores. We test LoQuM by comprehensive cross-validation studies on the human genome. The cross-validation studies are conducted by using different simulators to generate the training and testing data. Namely, we first simulate training reads using the Seal (Ruffalo *et al.*, 2011) sequencing simulation software, and we use validation reads generated by the ART (Huang *et al.*, 2012) software. We also investigate the utility of recalibrating mapping quality scores in improving genomic variant detection in the context of detecting SNPs. For this purpose, we implant SNPs into the human genome and use the SAMtools (Li *et al.*, 2009a) software to compare the effect of raw and recalibrated mapping quality scores to the performance of SAMtools in calling these implanted SNPs. Previous work such as SRMA (Homer and Nelson, 2010) focused on local re-alignment of short reads to improve the accuracy of SNP calls; LoQuM's broader focus also makes it suitable for improving the accuracy of other types of variant calls.

The results of our experimental studies can be summarized as follows:

- For most aligners, the mapping quality scores estimated by LoQuM strongly correlate with actual mapping accuracy.
- The quality scores computed by LoQuM provide a reliable criterion for selecting the high-quality mappings among those that are assigned zero quality scores or not scored at all by the aligner.
- The features that are most useful in assessing mapping quality are the raw mapping qualities provided by the alignment tools and (except for Novoalign) the rate of degradation of base quality for a read. For tools that report multiple mappings for reads (e.g. mrFAST), the number of mappings for a read is also highly informative.
- Mapping quality scores computed by LoQuM greatly improve the precision of SNP calling at the cost of moderately lower recall.

### 1.4 Outline

In the next section, we describe our classification framework for calibrating the quality of short read mappings. Subsequently, in Section 3, we present the results of comprehensive cross-validation studies performed on three different aligners (BWA, SOAP2 and Novoalign) using the human genome and the impact of these results on SNP calling. In Section 4, we interpret our findings, discuss how LoQuM can be useful in a range of applications and outline avenues for future research.

## 2 METHODS

**Fig. 2.**
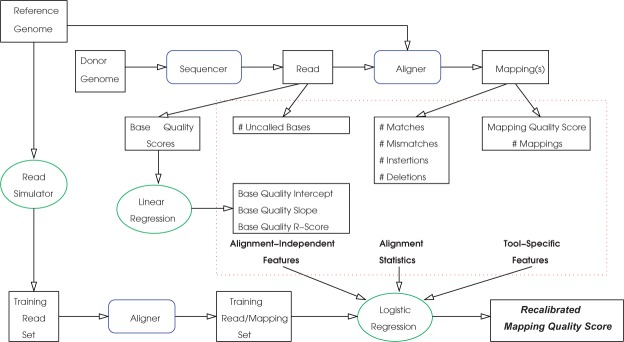
The machine learning framework for recalibrating mapping quality scores. Rectangles represent data, blue rounded rectangles represent available hardware and software and green ellipses represent computational methods implemented within LoQuM

The proposed machine learning framework is shown in Figure 2. As seen in the figure, the reads are generated by the sequencer from the donor genome and are aligned to the reference genome using an available alignment tool. Both the sequencer and the aligner provide certain statistics on the read and the alignment, which are used by LoQuM to recalibrate the mapping quality scores.

In the proposed framework, we first derive features for each read-mapping pair from the read statistics provided by the sequencer and the alignment statistics provided by the aligner. Subsequently, we generate a large set of reads for training, by simulating reads from the reference genome. We then feed these reads to the aligner to generate a set of training mappings. We derive classification features for the training read-mapping pairs based on the model used for generating reads and the alignment statistics. As we know exactly where each simulated read is supposed to be mapped on the reference genome, we label each read-mapping pair in the training set as ‘accurate’ or ‘not accurate’. Using this training data set, we fit a logistic regression model that represents the relationship between the accuracy of a mapping and the features derived from the read and alignment statistics. Finally, we use this logistic regression model to predict the accuracy of each mapping in the test dataset. As logistic regression makes a quantitative prediction for mapping accuracy between 0 and 1, we use this figure directly as a measure of mapping quality.

In this section, we first discuss the features that are used in assessing the quality of short read mappings. Subsequently, we describe the proposed classification framework for recalibrating mapping quality scores using these features.

### 2.1 Selection of Features

We classify the features we use into three categories: (i) alignment-independent features, (ii) alignment statistics and (iii) aligner-specific features. We discuss the features in each category later.

#### 2.1.1 Alignment-independent features

These features consist of read statistics reported by the sequencer. In particular, most types of sequencing hardware provide a quality score *Q*_b_ for each base call in the read. In other words, the hardware reports its confidence in assigning a specific nucleotide to each base (e.g. the base is a G with 99.9% confidence or a C with 80% confidence). The raw quality value, also called Phred score, is the log-scaled probability that the base call is incorrect:
(1)
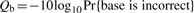


The base quality scores reported by sequencers range from 0 (least reliable) to 40 (most reliable).

**Fig. 3.**
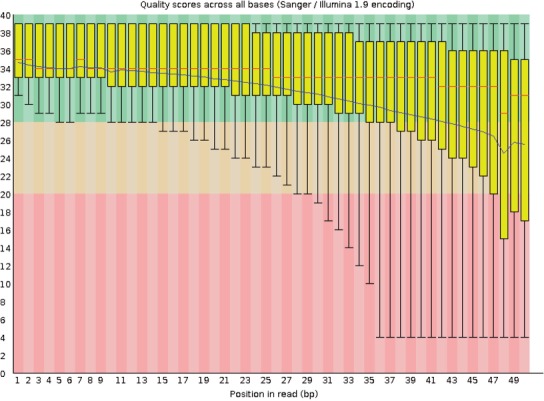
Boxplots of per-base quality statistics provided by the FastQC tool (Andrews, 2010) for the MDAMB468 cell line (Sun *et al.*, 2011) across a random sample of the 33 million reads. The *x*-axis is the base position in each read, and the *y*-axis is the base quality score *Q*_b_. The blue line shows the mean base quality

Figure 3 shows base quality statistics for a real RNA-seq data set. In the figure, the distribution of base quality across a random sample of 33 million reads is shown as a function of the position of the base on the read. As seen in the figure, mean base quality decreases in an almost linear fashion as more bases are read.

If we use base quality scores directly as features for classification, we would have *k* base quality features for a read of length *k*, causing problems with high dimensionality. An alternate approach is to use the mean base quality for a read; however, the mean does not capture the base position-dependent nature of base quality scores. For these reasons, we fit a linear regression model to each read's quality scores and use the regression parameters as features for classification:

- Intercept: The quality of the first base in the read.
- Slope: The rate of decline of base quality as more bases are sequenced on the read.
- *R* value (correlation coefficient): How well a line fits the read's base quality scores. A low *R* value may signify, e.g. a read whose base quality values show a very sharp drop and then ‘bottom out’ at 0 for the remainder of the read.

Another potentially useful alignment-independent feature is the number of bases that could not be called (*N* count) in each read. When the sequencing hardware cannot identify the base at a certain position, it reports an *N* (instead of A, C, G and T) for that base, along with a zero base quality score. The *N* count should correlate with base quality statistics, but this may not be completely captured in the linear regression parameters described earlier. An *N* in the middle of a read should cause a sharp downward spike in base quality, with quality scores more-or-less resuming their previous value immediately afterward.

#### 2.1.2 Alignment statistics

Alignment tools report a few standard values, including the number of matches, mismatches, insertions and deletions in a mapping. These statistics together provide a direct measure of how well a read is aligned to a position in the reference genome. We use the raw counts of each of these values as classification features.

#### 2.1.3 Aligner-specific features

Alignment tools typically report their output in the standard SAM (Li *et al.*, 2009a) file format, which defines a mapping quality field that aligners can use to signify whether an alignment is likely to be correct. The SAM format defines this mapping quality *Q*_m_ to be analogous to the base quality *Q*_b_
(2)
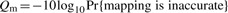


However, as discussed in the previous section, these mapping quality scores do not accurately characterize the reliability of a mapping. Among popular modern alignment tools, BWA and Novoalign provide the most meaningful quality scores (Ruffalo *et al.*, 2011), though we see that these tools still assign quality 0 to many accurate mappings (Fig. 1). As these quality scores are informative to a certain extent, we expect them to be useful as features for classification. However, we seek to improve on the false negatives—the accurate mappings that have low quality—and the false positives—the inaccurate mappings that have high quality.

Furthermore, some alignment tools (e.g. mrFAST and mrsFAST) may return multiple possible mappings for a read. The number of possible mappings for a read may be an indicator of the reliability of each of these mappings, as at most one of the mappings can correspond to the read's position in the genome. For this reason, for these tools, we also use the number of mappings returned for each read as a feature for classification.

### 2.2 Classification

We choose logistic regression as a classifier to learn the relationship between accuracy of mapping and the features derived in the previous section, for multiple reasons. First, as we are interested in accurately calculating the likelihood that a mapping is correct, a probabilistic classifier is a natural choice. Second, logistic regression makes no assumptions about the independence between features, which is appropriate in this setting. Many of the features described in Section 2.1 are not likely to be statistically independent of each other; at the very least, the mapping quality returned by the aligner should have some correlation with the other features. Finally, logistic regression is a simple classifier that provides insights on which features are most useful in predicting the dependent variable (here, accuracy of mapping), facilitating interpretation of how each feature affects mapping quality.

We seek to accurately model the probability *p* that a mapping is correct. Logistic regression represents this in terms of the log odds ratio 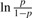 and models this quantity as a linear combination of numeric features *x*_i_ and coefficients *β_i_* (with a constant intercept term *β_0_*):
(3)
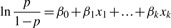


Our *x_i_* values are the features described earlier, e.g. mapping quality score, base quality slope and number of mappings.

### 2.3 Simulation of Reads

Our evaluation of LoQuM uses simulated 50 bp reads from the human genome, provided by the ART (Huang *et al.*, 2012) sequencing simulation package. ART uses empirical base quality profiles to accurately mimic the characteristics of Illumina hardware, and we use these base quality values to calibrate the machine learning framework. Each read is annotated with its original position in the reference genome, which allows for easy evaluation of whether an alignment tool mapped it to the correct location.

### 2.4 Cross-validation framework

We perform a standard 5-fold cross-validation procedure: we build the classifier on 80% of the training set and evaluate the accuracy on the remaining 20%. For demonstration purposes, figures in this document represent one of the five training/validation folds.

### 2.5 SNP calling evaluation

Our evaluation of SNP calling used 100 artificial SNPs inserted into random locations in human chromosome 1. We created a new reference sequence containing these SNPs, then simulated reads from the altered sequence with ART. We then aligned these reads to chromosome 1 with BWA and adjusted these reads' mapping quality scores with LoQuM. We identified significant SNPs with the SAMtools mpileupvariant calling program, both with BWA's original mapping quality scores and with LoQuM's recalibrated scores. Using the locations of the artificial SNPs, we evaluated the number of true-positive, false-positive and false-negative SNP calls with each set of mapping quality scores.

## 3 RESULTS

We evaluate our classification framework by considering the relationship between precision and recall as one varies a threshold on prediction output. In calculating these measures, we consider a true positive (TP) as a read that is correctly mapped and whose score exceeds this threshold. A false positive (FP) is a read that is incorrectly mapped but whose score exceeds this threshold, and a false negative (FN) is a read that is correctly mapped but is discarded because its score is less than the threshold. We then use the standard definition of precision as 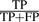. Similarly, recall is defined as 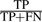.

We evaluate the performance of LoQuM on multiple alignment tools and compare the classifier output with the raw mapping quality (if provided). We then examine the relationship between our prediction scores and actual mapping accuracy and the effect of these recalibrated mapping quality scores on SNP calling with the SAMtools (Li *et al.*, 2009a) mpileupvariant caller.

### 3.1 BWA

**Fig. 4.**
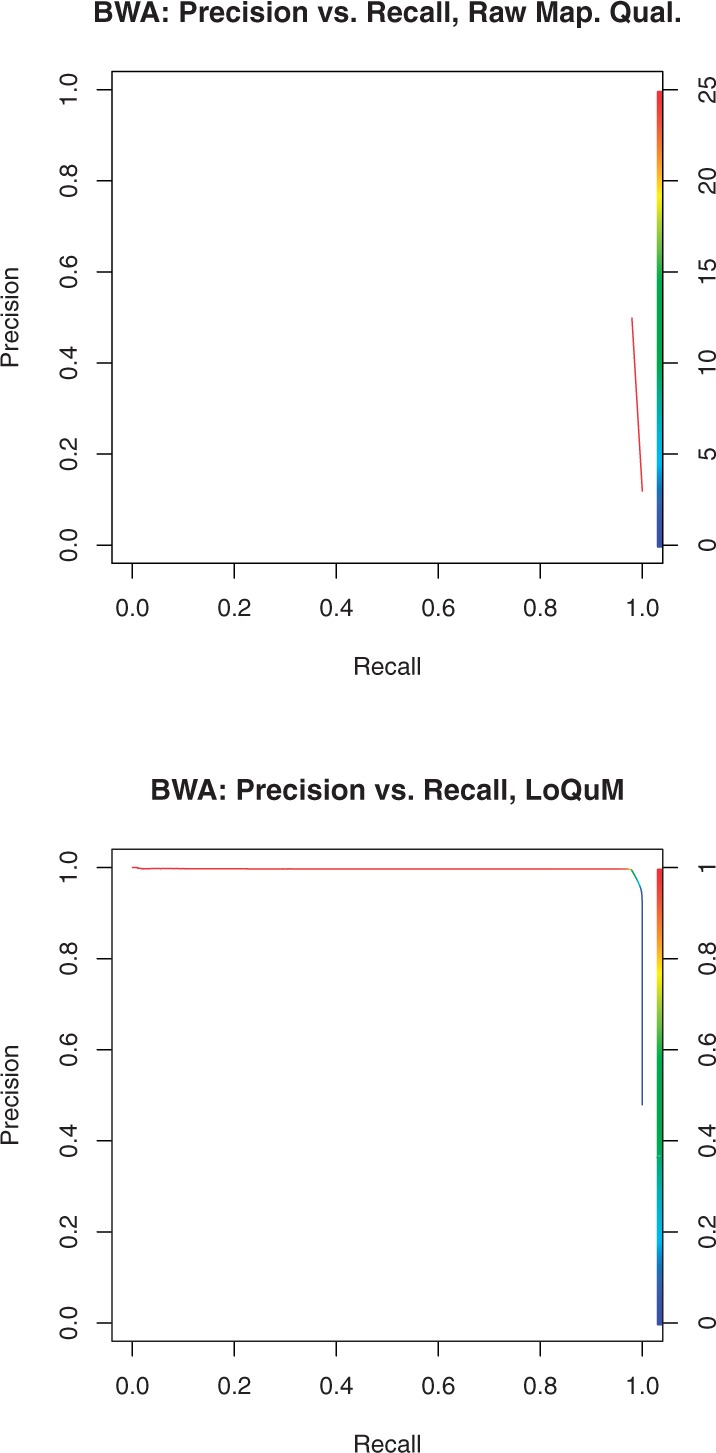
Precision versus recall for the alignment tool BWA, using the raw mapping qualities in (**a**), and the output of LoQuM in (**b**). The color of the curve denotes a threshold on mapping quality or prediction output; decreasing this threshold typically increases recall but decreases precision

Figure 4 shows precision versus recall for the BWA alignment tool, with Figure 4a showing the raw mapping qualities and Figure 4b showing the output of LoQuM. We see that the raw mapping qualities provide relatively little information about reads that are incorrectly mapped—even at the highest mapping quality of 25, BWA provides high recall and precision of ≈ 0.5. LoQuM, however, identifies these high-quality incorrect mappings and provides much higher precision at comparable recall values.

### 3.2 SOAP

**Fig. 5.**
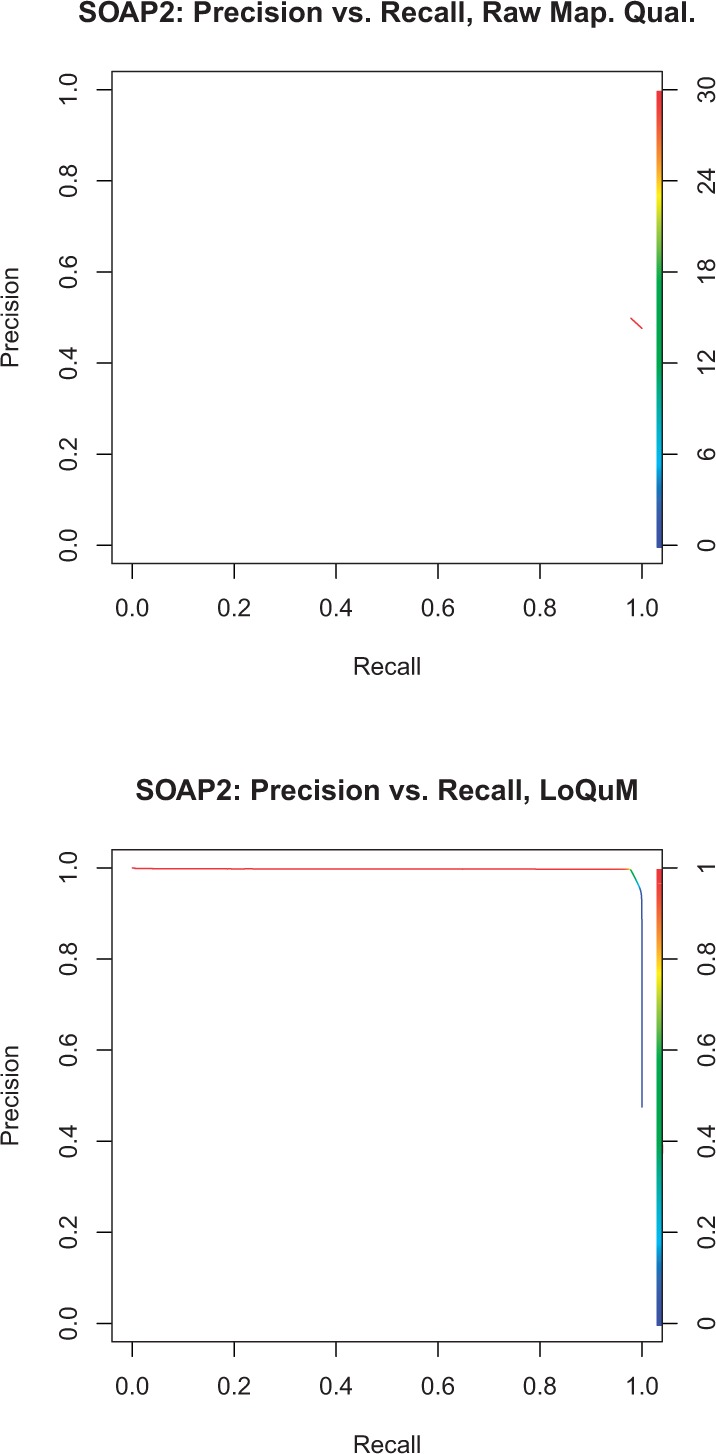
Precision versus recall for the alignment tool SOAP2, using the raw mapping qualities in (**a**), and the output of LoQuM in (**b**). The color of the curve denotes a threshold on mapping quality or prediction output; decreasing this threshold typically increases recall but decreases precision

Figure 5 shows SOAP2's precision and recall as a function of a threshold on mapping quality or logistic regression output. From the raw mapping quality precision/recall in Figure 5a, we see that SOAP's quality scores have very low dynamic range and discriminative power. In fact, SOAP only reports mapping qualities of 0 or 30, and all quality-30 mappings are at the top of the red line (precision≈ 0.5) with all quality-0 mappings represented as an invisible blue dot under the red line at precision 0.45. Nonetheless, this logistic regression framework can accurately classify reads as correctly or incorrectly mapped. This is shown in Figure 5b—LoQuM provides higher area under the precision/recall curve, and a score threshold of 0.5 sits at the ‘elbow’ of the curve (the point at which mappings become less trustworthy).

### 3.3 Novoalign

**Fig. 6.**
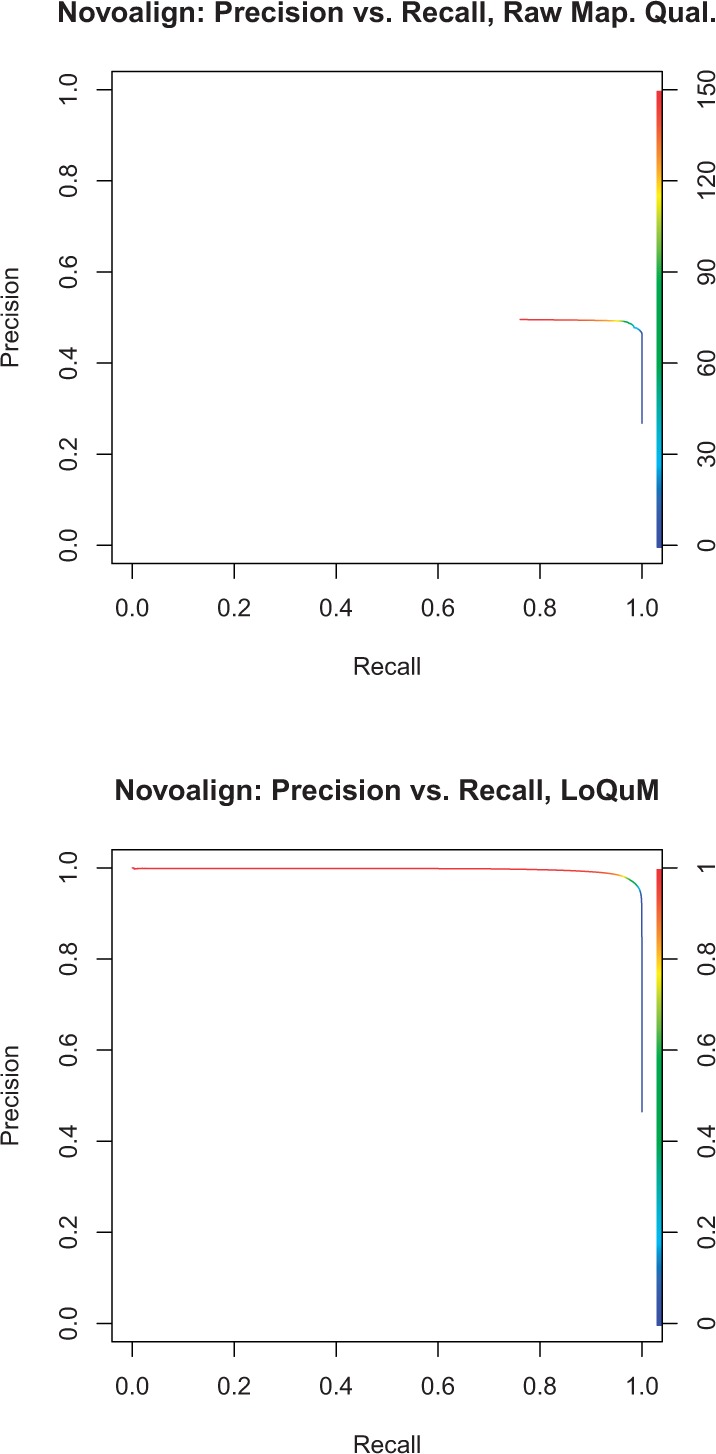
Precision versus recall for the alignment tool Novoalign, using the raw mapping qualities in (**a**) and the output of LoQuM in (**b**). The color of the curve denotes a threshold on mapping quality or prediction output; decreasing this threshold typically increases recall but decreases precision

Again, Figure 6 shows precision versus recall for Novoalign's mapping qualities and LoQuM's output. From Figure 6a, we see that Novoalign provides the most dynamic range in its mapping quality, though still with a maximum precision of ≈ 0.5. This logistic regression model produces a great accuracy improvement on the output of Novoalign; we see a smoothly decreasing curve in Figure 6b. This suggests that LoQuM captures a significant amount of information about Novoalign's mappings beyond what is represented in its quality scores.

### 3.4 Overall

**Fig. 7.**
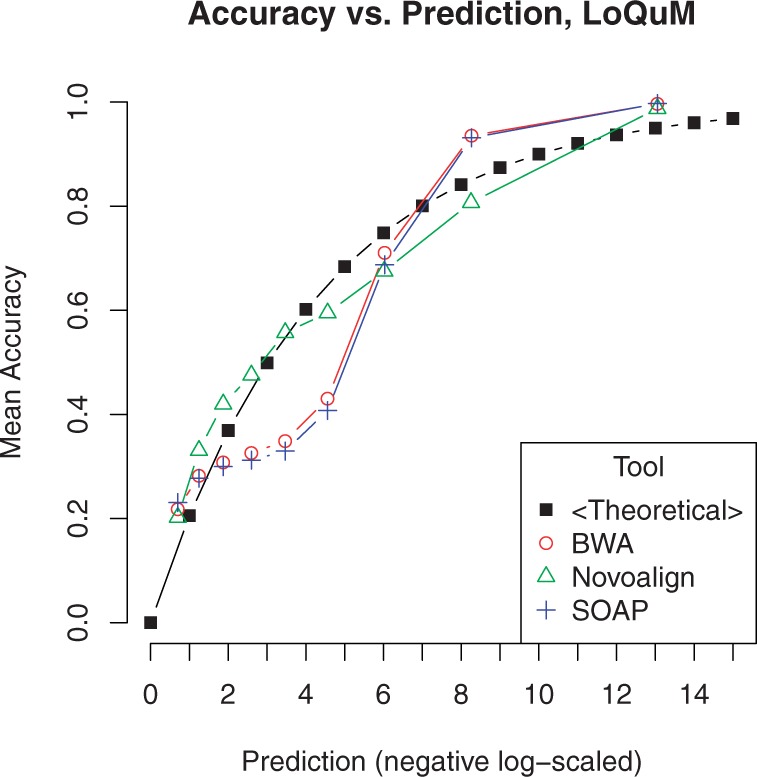
Comparison of reported accuracy versus theoretical accuracy for ART's simulated reads. The *x*-axis is the output of the logistic regression classifier *p* after inversion and negative log-scaling: *Q* = −10log_10_(1 − *p*). This corresponds to the mapping quality score *Q*_m_ in Equation (2)

Figure 7 shows similar data as 1 but uses the output of LoQuM instead of the raw mapping quality. Mappings are divided into 10 groups based on their prediction score, and the average accuracy for each group is plotted against the theoretical accuracy at that score. We see that LoQuM's output generally matches the theoretical accuracy much better than the raw mapping quality shown in Figure 1.

### 3.5 SNP calling

Our SNP calling results are listed in Table 1. We see that the SAMtools mpileup variant calls are dominated by false positives; raw mapping qualities provide a recall score of 0.83 but precision of only 0.163. The recalibrated mapping quality scores provided by LoQuM result in lower recall (0.63) but a substantially higher precision of 0.589. These results are consistent with the precision/recall curves shown above—alignment tools report many incorrect mappings with high mapping quality, and LoQuM's more conservative quality scores indeed reduce false-positive SNP calls.

**Table 1.** SNP calling results for 100 artificial SNPs inserted into human chromosome 1

	Raw Map. Qual.	LoQuM scores
True positives	83	63
False positives	427	44
False negatives	17	37
Precision	0.163	0.589
Recall	0.83	0.63
*F* score	0.136	0.304

The *F* score is defined as the harmonic mean of precision and recall: *F* = 1/(1/prec + 1/rec).

## 4 DISCUSSION

As shown in the above figures, this logistic regression model provides higher precision at various recall values. This framework also provides better dynamic range than the raw mapping quality values provided by most tools, suggesting that it may be valuable for researchers who desire more granularity in their data analysis.

Most importantly, LoQuM correctly identifies many correctly mapped reads with lower mapping quality. This is evident in the precision/recall plots; a classifier output threshold of 0.5 sits exactly at the ‘elbow’ of each curve (the point at which precision starts to significantly decrease). LoQuM's scores therefore provide a reliable way to discriminate between correct and incorrect mappings across different alignment tools—one may consistently use 0.5 as a quality score threshold. This suggests that researchers can use a tool of this type to use more of their data and get better yields out of sequencing experiments.

The differences in feature significance between alignment tool classifiers also highlight the characteristics of these tools and the information that is likely to be represented in their existing quality scores.

## 5 CONCLUSION

In this article, we show that a machine learning framework that uses a combination of read and alignment statistics as classification features and simulated reads as training data to predict mapping accuracy can improve on mapping quality scores reported by available tools. These improved mapping quality scores might be particularly useful in improving the identification of genomic variants, since they can ‘save’ potentially informative mappings that would be conservatively assigned low-quality scores by the assignment tools. Additionally, LoQuM's recalibrated mapping quality scores can reduce false-positive variant calls due to overestimated mapping accuracy. Assessment of the effect of mapping quality scores on identification of more genomic variants remains an important and interesting problem for future studies.

*Funding*: This work was supported by National Science Foundation Award IIS-0916102 and National Cancer Institute Grant No. R01CA131341.

*Conflict of Interest*: none declared.
